# Phase II trial of daratumumab with DCEP in relapsed/refractory multiple myeloma patients with extramedullary disease

**DOI:** 10.1186/s13045-022-01374-5

**Published:** 2022-10-23

**Authors:** Ja Min Byun, Chang-Ki Min, Kihyun Kim, Soo-Mee Bang, Je-Jung Lee, Jin Seok Kim, Sung-Soo Yoon, Youngil Koh

**Affiliations:** 1 Department of Internal Medicine, Seoul National University Hospital, Seoul National University College of Medicine, 101 Daehak-Ro, Jongno-Gu, Seoul, 03080 Korea; 2 Department of Hematology, Seoul St. Mary’s Hematology Hospital, College of Medicine, The Catholic University of Korea, Seoul, Korea; 3 Division of Hematology/Oncology, Department of Medicine, Sungkyunkwan University School of Medicine Samsung Medical Center, Seoul, Korea; 4 Department of Internal Medicine, Seoul National University Bundang Hospital, Seoul National University College of Medicine, Seongnam, Korea; 5 Chonnam National University Hwasun Hospital, Chonnam National University Medical School, Hwasun, Korea; 6 Division of Hematology, Department of Internal Medicine, Yonsei University College of Medicine, Severance Hospital, Seoul, Korea

**Keywords:** Multiple myeloma, Relapse/refractory, Extramedullary multiple myeloma, Daratumumab, DCEP

## Abstract

Extramedullary multiple myeloma (EMD) is an aggressive subentity of multiple myeloma (MM) with poor prognosis. As more innovative therapeutic approaches are needed for the treatment of MM with EMD, we conducted this multicenter, non-randomized phase II trial of daratumumab in combination with dexamethasone, cyclophosphamide, etoposide and cisplatin (DARA-DCEP). A total of 32 patients (median age 59, range 35–73) were treated with DARA-DCEP. Based on the best response during the study, the complete remission (CR) rate was 35.5% and overall response rate (ORR) 67.7%. During the median follow-up of 11 months, the median progression-free survival (PFS) was 5 months and median overall survival (OS) 10 months. There were 7 long-term responders whose median PFS was not reached. The most common grade ≥ 3 hematologic AE was thrombocytopenia. The most common non-hematologic AE was nausea (22.6%), followed by dyspepsia, diarrhea and stomatitis (all 12.9%). Grade ≥ 3 daratumumab infusion-related reaction was noted in 9.7% of the patients. Except for the planned 30% dose adjustment in cycle 1, only 2 patients required DCEP dose reduction. This is one of the very few prospective trials focusing on EMD and we successfully laid grounds for implementing immunochemotherapy in MM treatment.

**Supplementary Information:**

The online version contains supplementary material available at 10.1186/s13045-022-01374-5.

## Key points

1. This is one of the very few prospective trials focusing on refractory multiple myeloma with extramdedullary disease (EMD).
2. We successfully laid grounds for implementing immunochemotherapy in EMD treatment.

**Supplementary Information:**

The online version contains supplementary material available at 10.1186/s13045-022-01374-5.

## Introduction

Extramedullary multiple myeloma (EMD) is an aggressive subentity of multiple myeloma (MM), characterized by the ability of a subclone to grow outside of the bone marrow, resulting in a high-risk state associated with increased proliferation, evasion of apoptosis and treatment resistance. Despite the improvements in survival for most patients with MM over recent decades with development of newer immunomodulatory drugs (IMiDs) and proteasome inhibitors (PIs), treatment outcomes remain less than satisfactory for EMD [1, 2].

Interestingly, this is an area where traditional cytotoxic chemotherapy is still in effect. Although not robust, based on available data [3–7] lymphoma-like approach with polychemotherapy regimen followed by stem cell transplantation remains the current first-line approach. As such, even with increasing arsenal of therapeutic options in year 2022, VD-PACE (bortezomib, dexamethasone–cisplatin, doxorubicin, cyclophosphamide and etoposide) plus IMiD [7, 8] is the recommended primary therapy for EMD [9].

Unfortunately, for bortezomib refractory patients with EMD there is no standard of care. Daratumumab (DARA), the first human anti-CD38 IgG1 monoclonal antibody, has shown some promising efficacy in patients with EMD. In GEN501 [10] and SIRIUS [11] studies, daratumumab monotherapy showed responses across all subgroups including patients with extramedullary plasmacytoma and those with triple- or quadruple-refractory disease or high-risk cytogenetics. Collectively, the reported overall response rate (ORR) of daratumumab monotherapy for heavily pretreated MM patients with EMD is around 20% [11–16].

To address the unmet medical needs in treatment of relapsed/refractory (R/R) MM with EMD, we conducted this phase II study combining DARA with chemotherapy DCEP (dexamethasone, cyclophosphamide, etoposide and cisplatin). Considering (1) the success of immunochemotherapy in B-cell non-Hodgkin lymphoma treatment and (2) the clinical and morphological resemblances between lymphoma and mass-forming MM [17, 18], we hypothesized that DARA-DCEP can induce synergy to effectively control R/R MM with EMD. To mitigate the concerns regarding hematologic adverse events of cytotoxic chemotherapy, prophylactic granulocyte colony-stimulating factor (G-CSF) was used and liberal DCEP dose reduction was allowed.

## Methods

### Design overview

This open-label, multicenter, non-randomized phase II trial was carried out in 6 tertiary hospitals in Korea between August 2019 (first patient in after ClinicalTrials.gov registration) to January 2020 (ClinicalTrials.gov identifier: NCT04065308). The study was conducted according to the Declaration of Helsinki and was approved by the institutional review board of each hospital. Informed consent was taken from all patients before participating in any study-related procedure.

### Study population

Patients older than 19 years with MM with EMD relapsed/refractory to bortezomib according to the International Myeloma Working Group (IMWG) [19, 20] were considered eligible for enrollment. The definition of “EMD” was inclusive of (1) soft tissue masses in extra-osseous locations and (2) bone-related plasmacytomas that extend via disruption of cortical bones into contiguous soft tissues. Patients had to have measurable disease with short-axis diameter ≥ 1 cm on either CT (computed tomography) or PET-CT (^18^F-fluorodeoxyglucose positron emission tomography-CT) at enrollment.

Additionally, only the patients with ECOG (Eastern Cooperative Oncology Group) performance status of 0–2 and adequate bone marrow function, defined as absolute neutrophil count (ANC) ≥ 1.5 × 10^9^/L, platelet count ≥ 50 × 10^9^/L and hemoglobin ≥ 7.5 g/dL, were allowed to participate. Patients with creatinine clearance < 25 mL/min were excluded. Patients with non-secretory myelomas and current or history of plasma cell leukemia were also excluded. A washout period of 2 weeks prior to cycle 1 day 1 was required.

### Intervention

**Fig. 1:**
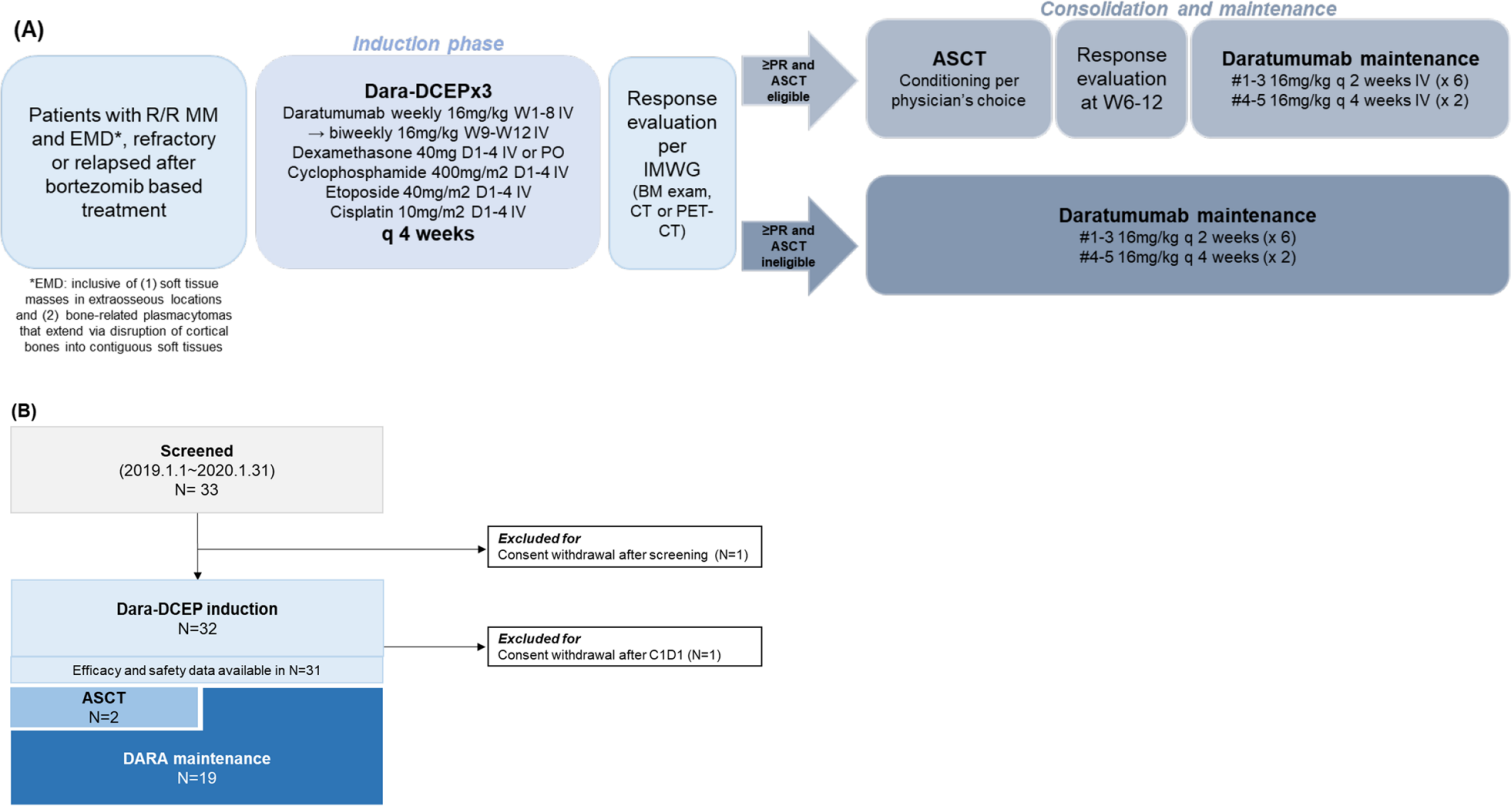
**A** Study overview. **B** CONSORT diagram

As shown in Fig. 1A, patients received 3 cycles of DARA-DCEP induction, followed by 5 cycles of DARA maintenance. During the induction phase, patients underwent a 28-day treatment cycle: dexamethasone 40 mg (either orally or intravenously), cyclophosphamide 400 mg/m^2^, etoposide 40 mg/m^2^ and cisplatin 10 mg/m^2^ on days 1–4. For DCEP, dose reduction up to 30% was allowed per attending physician’s decision from cycle 1. Dose delay was defined as a > 7-day delay for any subsequent cycle after cycle 1. Daratumumab was given at 16 mg/kg weekly from week 1–8, then 16 mg/kg biweekly from week 9–12. During the induction phase, patients were admitted and prophylactic pegteograstim 6 mg (Neulapeg®, GC Biopharma Corp.) was given 24 h after chemotherapy completion (day 6) each cycle. Either ciprofloxacin 500 mg twice daily or trimethoprim–sulfamethoxazole (80 mg trimethoprim and 400 mg sulfamethoxazole) twice daily was used as prophylactic antibiotics.

After 3 cycles of induction, bone marrow examination and imaging evaluation were done for response assessment. If partial response (PR) or better response was achieved per IMWG crtieria [19, 20], the patient was able to undergo either autologous stem cell transplantation (ASCT) followed by DARA maintenance or DARA maintenance depending on the circumstances. For patients undergoing ASCT, DARA maintenance had to be administered within 12 weeks of ASCT. During the maintenance phase, daratumumab was given at 16 mg/kg biweekly for 6 doses then 16 mg/kg monthly for 2 doses outpatient based.

### Endpoints and statistical analysis

The primary endpoint of the study was the complete response (CR) rate. The secondary endpoints included ORR, progression-free survival (PFS), overall survival (OS) and adverse events (AE).

The response evaluation was carried out according to the IMWG criteria [19]. EMD CR was confirmed by PET-CT in all cases. ORR was defined as PR or better response. The PFS was defined as time from study drug administration to relapse or death from any cause. The OS was defined from study drug administration to death of any cause. Patients were followed up until January 2022. The AE were assessed according to the National Cancer Institute Common Terminology Criteria for Adverse Events version 4.03.

The sample size was calculated based on Fleming single-stage procedure. The assumption was that if the CR rate was 35% [11] this would be considered effective. With a power of 80%, alpha = 5% and 10% dropout rate, 33 patients were required.

Fisher’s exact test was used for nominal variables, and Mann–Whitney U test was used for continuous variables. The survival curves were estimated using the Kaplan–Meier method. All data were analyzed using the Statistical Package for the Social Sciences software (IBM^®^ SPSS^®^Statistics, version 25.0).

## Results

### Baseline characteristics

A total of 33 patients were enrolled, but 1 patient withdrew consent after screening (Fig. 1B). The baseline characteristics of 32 patients who received at least 1 dose of study drugs are shown in Table 1. There were 23 patients (71.9%) who had soft tissue masses in extra-osseous locations with or without bone lesions and 9 patients (28.1%) who had only bone-related plasmacytomas. The most common site of extramedullary disease was chest (Additional file 1: Table 1). Risk stratification was available in 23 patients: 16 out of 23 (69.6%) were identified as high risk. There were 4 patients (17.4%) who harbored del (17p) and 6 patients (26.1%) with t(4;14) and t(14;16) each. Median prior lines of therapy was 3 (range 1–5), and median time to study screening from MM diagnosis was 26 months (range 3–101). All patients were exposed to bortezomib, 78.1% to carfilzomib and 90.6% to lenalidomide prior to study enrollment. Twenty-seven (84.4%) were double-refractory patients. Of the 19 patients who had previously undergone ASCT, all but 4 underwent melpahalan 200 mg/m^2^ conditioning: 3 received busulfan-melphalan (BuMEL) conditioning while 1 received thiotepa-busulfan-cyclophosphamide (TBC) conditioning.

**Table 1:** Baseline characteristics

Characteristics	Total (*N* = 32)
Age at diagnosis, years, median (range)	56 (34–71)
Age at clinical trial screening, years, median (range)	59 (35–73)
Diagnosis to clinical trial screening, months, median (range)	26 (3–101)
Male, *N* (%)	22 (68.8)
Extramedullary disease at diagnosis, *N* (%)	18 (56.3)
Extramedullary disease at enrollment, *N* (%)	32 (100.0)
ISS at diagnosis, *N* (%)	
I/II/III	7 (21.9)/ 14(43.7) / 11(34.4)
R-ISS at diagnosis, *N* (%)	
I/II/III/unknown	3 (9.4)/ 7(21.9)/ 13(40.6) /9(28.1)
High risk, *N* (%)	Available in 23 patients 16/23 (69.6%)
t(4;14)	6/23 (26.1)
del(17p)	4/23 (17.4)
t(14;16)	6/23 (26.1)
Number of prior lines of treatment, median (range)	3 (1–5)
Prior ASCT, *N* (%)	19 (59.4)
Prior proteasome inhibitor use, *N* (%)	
Bortezomib	32 (100)
Carfilzomib	25 (78.1)
Ixazomib	2 (6.3)
Prior IMiDs use, *N* (%)	
Thalidomide	25 (78.1)
Lenalidomide	29 (90.6)
Pomalidomide	14 (43.8)
Prior alkylator exposure, *N* (%)	16 (50.0)
Refractory to proteasome inhibitor, *N* (%)	28 (84.5)
Refractory to IMiDs, *N* (%)	30 (93.8)
Double refractory, *N* (%)	27 (84.4)
ECOG performance at enrollment, *N* (%)	
0/1/2	7(21.9) / 19(59.4)/ 6(18.8)
Laboratory findings at enrollment, mean (standard deviation)	
Hemoglobin, g/dL	13.5 (1.8)
WBC, × 10^9^/L	5.7 (5.2)
ALC, × 10^9^/L	1.5 (1.5)
ANC, × 10^9^/L	2.8 (1.6)
Platelet, × 10^9^/L	171.7 (104.6)

*ISS* International Staging System; *R-ISS* Revised International Staging System; *ASCT* autologous stem cell transplantation; *IMiDs* immunomodulatory drugs; *ECOG* Eastern Cooperative Oncology Group; *ALC* absolute lymphocyte count; *ANC* absolute neutrophil count

### Treatment outcomes

Overall, 25 patients (78.1%) were able to complete 3 cycles of DARA-DCEP and 7 (21.9%) completed all 8 cycles (3 cycles of DARA-DCEP + 5 cycles of DARA maintenance). There were 3 patients who were taken off the trial for additional treatment: 1 after completing 3 cycles of DARA-DCEP for radiation therapy, 1 after completing 3 cycles of DARA-DCEP for allogeneic stem cell transplantation and 1 after completing 3 cycles of DARA maintenance for delayed autologous stem cell transplantation.

One patient withdrew consent after cycle 1 day 1, thus treatment outcomes and toxicity profiles were evaluated in 31 patients. Based on the best response during the study, the CR rate was 35.5% (11/31) and ORR was 67.7% (Table 2). There were no differences between the soft tissue plasmacytoma patients (39.1%) versus bone-related disease only (25.0%) with regard to the CR rate (*p* = 0.472). There were 2 patients who were serological CR but persistent EMD; thus, their responses were classified as VGPR.

**Table 2:** Treatment outcomes

*N*, (%)	Total
Study completion	
Cycle 3	25/32 (78.1)
Cycle 8	7/32 (21.9)
Reasons for no completing the study	
Progression	19/25 (76.0)
Death	2/25 (8.0)
Others	4/25 (16.0)
Consent withdrawal	1
For additional treatment	3
Best response during the study	
sCR + CR	11/31 (35.5)
VGPR	3/31 (9.7)
PR	7/31 (22.6)
SD	7/31 (22.6)
PD	3/31 (9.7)
ORR (sCR + CR + VGPR + PR)	21/31 (67.7)
NE	1
DCEP dose reductions	
Cycle 1 (*N* = 32)	Full dose 9/32 (28.1%); planned 30% DR 23/32 (71.9%)
Cycle 2 (*N* = 29)	Same dose as Cycle 1 25/29 (86.2%); dose reduction from Cycle 1 2/29 (6.9%)*; dose escalation from Cycle 1 2/29 (6.9%)
Cycle 3 (*N* = 25)	Same dose as Cycle 2 25/25 (100%)
DCEP dose delays	
Cycle 2 (*N* = 29)	4 (13.8)
Cycle 3 (*N* = 25)	3 (12.0)
ASCT during clinical trial	2/32 (6.3)
History of prior ASCT	1
Infused CD34 cells, × 10^6^/kg, median (range)	4.53 (3.3.41–5.65)
Conditioning regimen	
Melphalan 200 mg/m^2^	2

*sCR* stringent complete response; *CR* complete response; *VGPR* very good partial response; *PR* partial response; *SD* stable disease; *PD* progressive disease; *NE* not evaluable; *DCEP* dexamethasone–cyclophosphamide–etoposide–cisplatin; *ASCT* autologous stem cell transplantation

*2 patients underwent additional dose reduction from Cycle 1

**Fig. 2:**
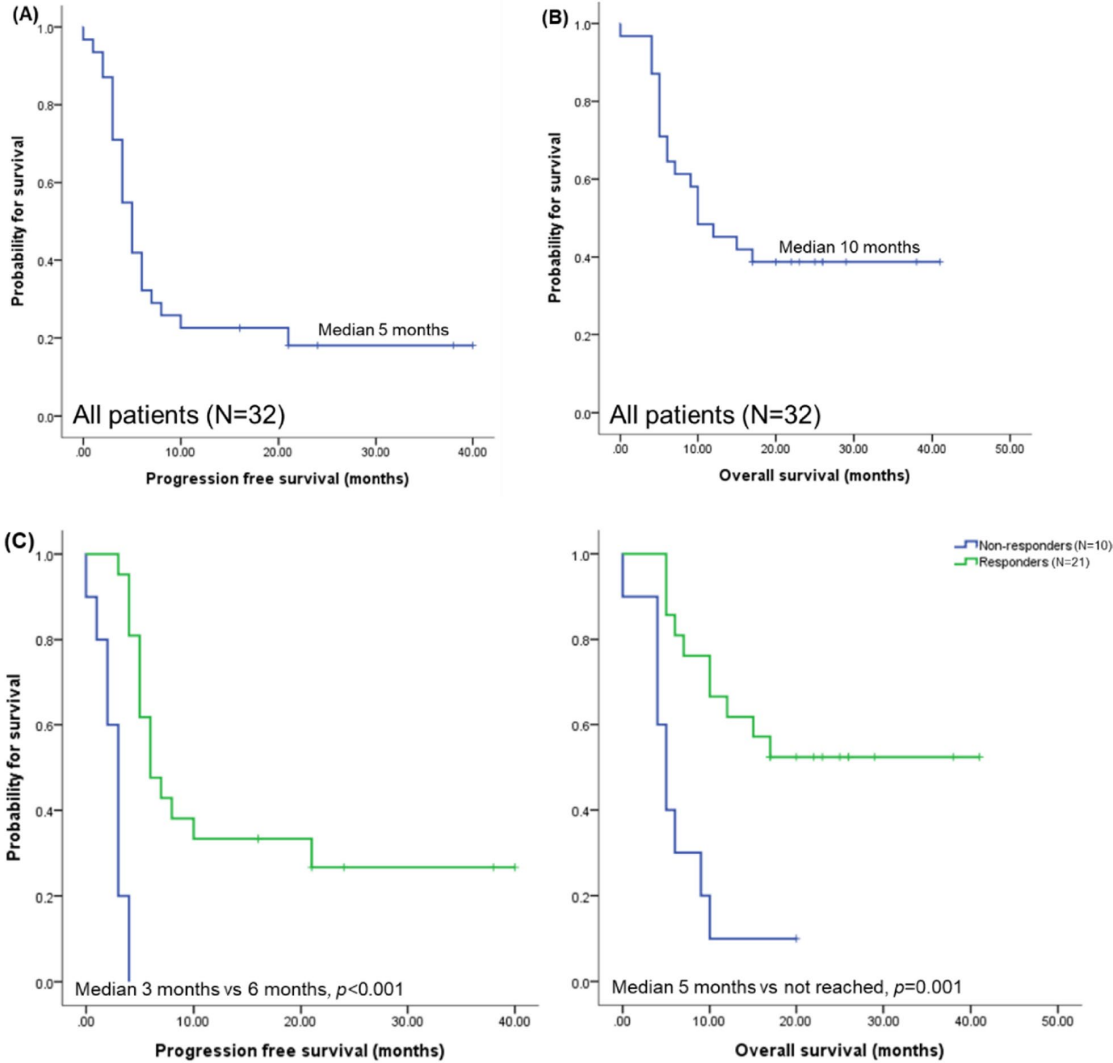
Survival outcomes. **A** Progression-free survival (PFS) of all patients. **B** Overall survival (OS) of all patients. **C** PFS and OS of non-responders (*N* = 10) versus responders (*N* = 21)

During the median follow-up of 11 months, the median PFS was 5 months (Fig. 2A) and median OS was 10 months (Fig. 2B). When patients achieving PR or better response (i.e., “responders”) were compared to those who did not (i.e., “non-responders”), responders showed significantly better PFS (median 6 months vs 3 months, respectively, *p* < 0.001) and OS (median not reached vs 5 months, *p* = 0.001) (Fig. 2C). Earlier exposure to DARA-DCEP showed trends toward better survival, but the difference did not reach statistical significance (DARA-DCEP as second line treatment vs third line or beyond, median PFS 6 months vs 5 months respectively, *p* = 0.233, data not shown). The 7 patients who completed the study, all but 1 remained in remission until data cutoff. For the 1 patient who progressed, the PFS was 10 months. The details of these long-term responders are summarized in Additional file 1: Table 2.

### Hematologic adverse events

In cycle 1, 9/32 (28.1%) patients received full dose of DCEP and 23/32 (71.9%) received 30% reduced dose per protocol (Table 2). There were 2 patients who underwent dose reduction in cycle 2, with final administered dose being 50% of the full dose DCEP. On the other hand, there were 2 patients who underwent dose escalation to receive full dose of DCEP for cycle 2, after receiving 30% reduced dose for cycle 1. No DCEP dose adjustment was needed for cycle 3.

There were 4 cases of dose delay for DARA-DCEP cycle 2, all due to cytopenias (Table 2). There were 3 cases of dose delay for DARA-DCEP cycle 3, 2 due to cytopenias and 1 due to general condition.

Hematologic adverse events are shown in Table 3. Thrombocytopenia was the most notable adverse events, with increasing incidence with cumulative chemotherapy cycles. During DARA-DCEP cycle 3, 36% of the patients experienced ≥ grade 3 thrombocytopenia. During DARA-DCEP cycle 1, 1 patient was treated for bacterial pneumonia and 1 patient for *Klebsiella pneumoniae* bacteremia. During DARA-DCEP cycle 2, there were 6 patients with documented infections: 1 pneumonia, 2 *Staphylococcus aureus* bacteremia, 2 disseminated herpes zoster infection and 1 localized herpes zoster infection. During DARA maintenance cycle 1, 1 patient had localized herpes zoster infection.

**Table 3:** Hematologic adverse events

	Any, *N* (%)	Grade ≥ 3, *N* (%)
DARA-DCEP cycle 1 (*N* = 31)		
Thrombocytopenia	8 (25.8)	5 (16.1)
Lymphopenia	7 (22.6)	2 (6.5)
Neutropenia	6 (19.4)	4 (12.9)
Documented infection	2 (6.5)	NA
DARA-DCEP cycle 2 (*N* = 29)		
Thrombocytopenia	8 (27.6)	5 (17.2)
Lymphopenia	5 (17.2)	4 (13.8)
Neutropenia	6 (20.7)	4 (13.8)
Documented infection	6 (20.7)	NA
DARA-DCEP cycle 3 (*N* = 25)		
Thrombocytopenia	12 (48.0)	9 (36.0)
Lymphopenia	9 (36.0)	5 (20.0)
Neutropenia	6 (24.0)	2 (8.0)
Documented infection	2 (8.0)	NA
DARA maintenance cycle 1 (*N* = 19)		
Thrombocytopenia	4 (21.1)	4 (21.1)
Lymphopenia	2 (10.5)	1 (5.3)
Neutropenia	0	0
Documented infection	1 (5.3)	NA
DARA maintenance cycle 2 (*N* = 14)		
Thrombocytopenia	0	0
Lymphopenia	1 (7.1)	1 (7.1)
Neutropenia	0	0
Documented infection	0	NA
DARA maintenance cycle 3 (*N* = 11)		
Thrombocytopenia	1 (9.1)	1 (9.1)
Lymphopenia	1 (9.1)	1 (9.1)
Neutropenia	1 (9.1)	1 (9.1)
Documented infection	0	NA
DARA maintenance cycle 4 (*N* = 9)		
Thrombocytopenia	0	0
Lymphopenia	0	0
Neutropenia	2 (22.2)	0
Documented infection	0	NA
DARA maintenance cycle 5 (*N* = 7)		
Thrombocytopenia	1 (14.3)	0
Lymphopenia	0	0
Neutropenia	0	0
Documented infection	0	NA

*NA* not applicable

During DARA-DCEP cycle 1, 5 patients (16.1%) were admitted for AE management: 2 for infection, 2 for neutropenic fever without documented infection and 1 for gastrointestinal (GI) bleeding. During DARA-DCEP cycle 2, 3 patients (10.3%) were hospitalized for AE management: 2 for infection and 1 for GI bleeding (same patient as cycle 1). During DARA-DCEP cycle 3, no patients were admitted for AE management.

### Non-hematologic adverse events

Daratumumab infusion-related reaction (IRR) was noted in 48.4% of the patients during the first cycle of DARA-DCEP. Three patients had grade 3 IRR (9.7%) with symptomatic bronchospasm, but recovered without sequelae. These 3 patients received prophylactic acetaminophen, H2-blocker and steroids for subsequent doses: 1 patient experienced grade 1 IRR during cycle 2 while other 2 patients did not have any more IRR events.

As shown in Table 4, the most common non-hematologic adverse event was nausea (22.6%) followed by dyspepsia (12.9%), diarrhea (12.9%) and stomatitis (12.9%). One patient developed deep vein thrombosis at axillary vein during DARA-DCEP cycle 2, which was treated with rivaroxaban.

**Table 4:** Non-hematologic adverse events

	Any, *n* (%)	Grade ≥ 3, *n* (%)
Daratumumab IRR		
Cycle 1 (*N* = 31)	15 (48.4)	3 (9.7)
Cycle 2 (*N* = 29)	1 (3.4)	0
Cycle 3 (*N* = 25)	0	0
During DARA consolidation	0	0
Nausea	7 (22.6)	1 (3.2)
Dyspepsia	4 (12.9)	0
Diarrhea	4 (12.9)	0
Stomatitis	4 (12.9)	0
Constipation	3 (9.7)	0
Hypertension	3 (9.7)	1 (3.2)
Anorexia	2 (6.5)	0
Peripheral sensory neuropathy	2 (6.5)	0
Hyperglycemia	1 (3.2)	1 (3.2)
Treatment related death	1 (3.1)	NA

*IRR* infusion-related reaction; *NA* not applicable

There were 2 deaths during the study (Table 2). One died due to underlying MM progression, while the other patient died due to combined bacterial and cytomegalovirus pneumonia. The latter patient was classified as treatment related mortality since the patient was in PR at the time of event (Table 4).

## Discussion

In this study, we combined daratumumab with conventional chemotherapy DCEP for the treatment of R/R MM with EMD and report ORR of 67.7% (21/31) with durable remission in 19.4% (6/31) of the treated patients. The importance of this trial lies on that 1) this is one of the very few prospective trials focusing on EMD and 2) we successfully laid grounds for implementing immunochemotherapy in MM treatment.

Despite its increasing incidence [21], the EMD responses in clinical trials have not been extensively analyzed thus there is no evidence-based consensus on the standard of care of EMD. The most validated modality is radiotherapy, but beyond radiotherapy patients are treated with multiple agents as per lymphoma treatment. DCEP regimen has been around for a while as a salvage treatment option for R/R MM patients, especially as bridge to high-dose therapy and stem cell transplantation or as a means of rapid tumor debulking [22–27]. The reported ORR of DCEP ranges from 45 to 58%, and more specifically, Park et al. reported ORR of 44.4% in Korean R/R MM with EMD (Additional file 1: Table 3). Daratumumab, on the other hand, is a more novel immunotherapeutic agent with reported ORR of 29.2% to 42.1% when used as monotherapy (Additional file 1: Table 3). When combined, we saw that DARA-DCEP indeed work synergistically as hypothesized, bringing the ORR up to 67.7%.

Cytopenia is always a concern when it comes to cytotoxic chemotherapy, especially in relapsed/refractory setting where patients have already been exposed to multiple lines of bone marrow damaging therapies. As such, we recorded the hematologic adverse events for each cycle (Table 3). The most prominent hematologic adverse event was thrombocytopenia in our study, with the incidence going up from 25.8% in cycle 1 to 48.0% in cycle 3. The reported incidence of thrombocytopenia with DCEP ranges from 62% to 76.3% [23, 27] and 21.6% to 57.9% with daratumumab monotherapy [11, 12, 16]. In the more recent CASSIOPEIA trial, in which daratumumab was combined with VTD (bortezomib–thalidomide–dexamethasone), thrombocytopenia was noted in 20% of the patients. Although direct comparisons are not possible, based on the numerical values, it is safe to surmise that DARA-DCEP combination treatment did not lead to increased toxicities. However, it should be noted that majority of the physicians chose to reduce the DCEP dose by 30% from the start, thus only 28.1% of the patients received full planned dose. Fortunately, the incidence of lymphopenia did not increase with prolonged daratumumab exposure: only 2 cases of lymphopenia were documented during the DARA maintenance phase. Also, with primary prophylactic granulocyte colony-stimulating factor (G-CSF) use, the incidence of ≥ grade 3 neutropenia ranged from 8% to 13.8% during DARA-DCEP induction. This is significantly lower than the reported incidence of ≥ grade 3 neutropenia from previous studies on DCEP (29.0% to 91.5%) [23, 27] and comparable to that from daratumumab monotherapy (10.1 to 35.5%) [11, 12, 16].

Another interesting point is that all of the attending physicians chose PET-CT over CT as means of EMD response evaluation. This is not surprising as PET-CT is considered the more suitable assessment tool of metabolically active EMD [28], and recent studies have demonstrated the negative correlation between abnormal PET-CT results and patient outcomes [29]. It is important to note that all EMD response evaluation in this study was corroborated by PET-CT results.

One of the most obvious limitations of this study is the small number of patients enrolled. We could not identify the prognostic or predictive factors related to DARA-DCEP response, including the role of consolidative ASCT as there were only 2 underwent ASCT during the clinical trial. Also, we were not able to clearly separate patients with soft tissue masses in extra-osseous locations versus those with bone-related plasmacytomas that extend via disruption of cortical bones. However, it should be taken into consideration that conducting a clinical trial in this certain setting is not easy, as evident by paucity of previous data. Also, with the advent of more potent immunotherapies, such as bispecific/trispecific antibodies and chimeric antigen T-cell (CAR T-cell) therapy, cytotoxic chemotherapy backbone might not seem very attractive to some. However, these newer immunotherapies are not without faults. While it is true that ide-cel and cilta-cel is the current go-to option for the treatment of adult patients with RR MM following 4 or more prior lines of therapy [30, 31], the production turnaround time of median 1 month calls for adequate bridging therapy. We believe DARA-DCEP can serve as a good bridging therapy especially because daratumumab targets CD38. Second pitfall of CAR T-cell therapy for EMD is the seemingly higher rates of adverse events [32] and shorter duration of response compared to cases without EMD [33]. Cytoreduction via DARA-DCEP bridging therapy could potentially be beneficial for decreasing cytokine release syndrome. Also, in the sense of minimal residual disease negativity conversion, DARA-DCEP may aid tumor control prior to CAR T-cell therapy infusion. Moreover, it should be noted that MM treatment is highly dependent on health-care resource distributions and thus vary greatly across the globe. Creative yet cost-effective combination such as DARA-DCEP can close such gaps while avoiding economic and/or regulatory constraints. Lastly, as polychemotherapy regimen is the incumbent “recommended” approach [7–9], our study results stand relevant.

All in all, DARA-DCEP is an effective regimen for controlling R/R MM with EMD after bortezomib failure. Adequate dose adjustments and primary prophylactic G-CSF use can maximize efficacy while minimizing toxicities.

## Supplementary Information

**Additional file 1**. **Supplementary Table 1.** Sites of extramedullary disease. **Supplementary Table 2.** Characteristics of 7 long-term responders. **Supplementary Table 3.** Comparison with previous studies.

## Data Availability

All data generated or analyzed during this study are included either in this article or in additional files.
