# Supplementary material for: Phase II trial of daratumumab with DCEP in relapsed/refractory multiple myeloma patients with extramedullary disease
Source: J Hematol Oncol. 2022 Oct 23;15:150. doi: 10.1186/s13045-022-01374-5 (PMC9590218; doi:10.1186/s13045-022-01374-5)
Supplement: Supplementary file 1 — Additional file 1. Supplementary Table 1. Sites of extramedullary disease. Supplementary Table 2. Characteristics of 7 long-term responders. Supplementary Table 3. Comparison with previous studies. [file 13045_2022_1374_MOESM1_ESM.docx]

**Supplementary Materials**

**Index**

Supplementary Table 1 ------------------------------------------------------------------------ Page 2

Supplementary Table 2 ------------------------------------------------------------------------ Page 3

Supplementary Table 2 ------------------------------------------------------------------------ Page 4

**Supplementary Table 1.** Sites of extramedullary disease

| **Anatomic site** | **N** |
| --- | --- |
| **Head & neck** | **5** |
| Central nervous system / skull | 2 |
| Oral cavity | 2 |
| Lymph nodes | 1 |
| **Chest** | **20** |
| Chest wall | 11 |
| Breast | 1 |
| Lung | 3 |
| Pleura | 5 |
| **Abdomen & pelvis** | **7** |
| Liver | 3 |
| Adrenal gland | 1 |
| Lymph nodes | 3 |
| **Skin/soft tissue** | **5** |
| **Skeletal muscle** | **3** |
| **Paraspinal area** | **5** |

**Supplementary Table 2.** Characteristics of 7 long-term responders

| **Index** | **Type** | **Pre-trial treatment** | **Trial treatment schema** |
| --- | --- | --- | --- |
| Patient #1 72/M | IgA lambda, CK | VMP#7→ixazomib maintenance→ DARA-DCEP | DARA-DCEP#3→maintenance, not relapsed, PFS 40 mo |
| Patient #2 73/M | IgG kappa | VMP#5→Rd#11→Kd#2→Pd#8→DARA-DCEP | DARA-DCEP#3→maintenance, not relapsed, PFS 38 mo |
| Patient #3 55/M | Kappa | VTd#1→ KRd#4→ ASCT→ re-KRd#3→PCd#6→DARA-DCEP | DARA-DCEP#3→maintenance, relapsed, PFS 10 mo |
| Patient #4 49/M | IgA kappa | VTd#3→ASCT→lenalidomide maintenance →DARA-DCEP | DARA-DCEP#3→maintenance, not relapsed, PFS 16 mo |
| Patient #5 54/M | IgG kappa | VTd#4→KRd#4→DARA-DCEP | DARA-DCEP#3→ASCT→maintenance, not relapsed, PFS 21 mo |
| Patient #6 61/M | IgA kappa, t(14;16)^*^ | Td#4→ASCT→Vd#1→Rd#26→PCd#10 →DARA-DCEP | DARA-DCEP#3→maintenance, not relapsed, PFS 24 mo |
| Patient #7 49/M | IgD lambda, CK | VTd#3→KRd#3→VAD#3→DARA-DCEP | DARA-DCEP#3→ASCT→maintenance, not relapsed, PFS 16 mo |

*Per fluorescence in situ hybridization (FISH)

M, male; CK, complex karyotype; VMP, bortezomib-melphalan-prednisone; mo, months; Rd, lenalidomide-dexamethasone; Kd, carfilzomib-dexamethasone; Pd, pomalidomide-dexamethasone; KRd, carfilzomib-lenalidomide-dexamethasone; ASCT, autologous stem cell transplantation; PCd, pomalidomide-cyclophosphamide-dexamethasone; VTd, bortezomib-thalidomide-dexamethasone; Td, thalidomide-dexamethasone; Vd, bortezomib-dexamethasone; VAD, vincristine-doxorubicin-dexamethasone ; PFS, progression free survival

**Supplementary Table 3.** Comparison with previous studies

|  | **Regimen** | **Setting** | **ORR** | **PFS** |
| --- | --- | --- | --- | --- |
| **Current** | DARA-DCEP | RR MM c EMD (N=31) | 67.7% | 5.0 months |
| **Park et al. (22)** | DCEP | RR MM (N=51) | 45.1% | 3.7 months |
|  |  | RR MM c EMD (N=9) | 44.4% | Unknown |
| **Griffin et al. (26)** | DCEP | RR MM (N=52)  *EMD in 15 (29%) | 51.9% | 3.8 months |
| **Lonial et al. (10)** | Daratumumab | RR MM (N=106) | 29.2% | 3.7 months |
|  |  | RR MM c EMD (N=14) | 21.4% | Unknown |
| **Usmani et al. (11)** | Daratumumab | RR MM (N=148)  *EMD in 18 (12%) | 31.1% | 4.0 months |
| **Park et al. (15)** | Daratumumab | RR MM (N=107)  *EMD in 22 (20.6%) | 42.1% | 3.6 months |
| **Minarik et al. (13)** | Daratumumab | RR MM (N=14)  *EMD in 3 (21.4%) | 38.5% | 4.6 months |

ORR, overall response rate; PFS, progression free survival; RR, relapsed/refractory; MM, multiple myeloma; EMD, extramedullary disease
